# Oral neural tumors: Clinicopathologic analysis of 157 cases and review of the literature

**DOI:** 10.4317/jced.55944

**Published:** 2019-08-01

**Authors:** Paris Tamiolakis, Evanthia Chrysomali, Alexandra Sklavounou-Andrikopoulou, Nikolaos G. Nikitakis

**Affiliations:** 1 DDS, MSc, Department of Oral Medicine and Pathology, Department of Dentistry, School of Health Sciences, National and Kapodistrian University of Athens, Greece; 2 DDS, PhD, Associate Professor, Department of Oral Medicine and Pathology, Department of Dentistry, School of Health Sciences, National and Kapodistrian University of Athens, Greece; 3 DDS, MSc, PhD, Professor, Department of Oral Medicine and Pathology, Department of Dentistry, School of Health Sciences, National and Kapodistrian University of Athens, Greece; 4 MD, DDS, PhD, Professor, Head of Department of Oral Medicine and Pathology, Department of Dentistry, School of Health Sciences, National and Kapodistrian University of Athens, Greece

## Abstract

**Background:**

Oral neural tumors (ONTs) are rare lesions and represent reactive or neoplastic proliferations of nerve sheath cells. The purpose of the present study is to report the clinical, demographic and histopathologic features of 157 ONTs diagnosed in a single Oral Pathology Department and review the pertinent literature.

**Material and Methods:**

157 cases of ONTs diagnosed during a 44-year period were retrospectively collected and the diagnosis was reconfirmed by studying representative haematoxylin and eosin stained tissue sections. The patients’ gender and age, as well as the main clinical features of the lesions, were retrieved from the biopsy submission forms.

**Results:**

The 157 ONTs represented approximately 0.4% of 35,590 biopsies accessioned during the study period. They affected 71 male and 86 female patients with a mean age of 38.4±18.8 years. They mainly appeared as asymptomatic nodules of normal or white colour on the tongue, lip mucosa and hard palate. The most common ONT was granular cell tumour (38.9%) followed by neurofibroma (19.7%), schwannoma (15.9%), traumatic neuroma (15.9%), palisaded encapsulated neuroma (8.3%) and nerve sheath myxoma (1.3%).

**Conclusions:**

This study confirmed the rarity of ONTs. Their clinical characteristics mimic other oral lesions; thus, microscopic examination is the only mean to arrive at a definitive diagnosis.

** Key words:**Oral neural tumors; oral neural neoplasms; neurofibroma; oral neurofibroma; schwannoma; oral schwannoma; neurilemmoma; oral neurilemmoma; granular cell tumor; oral granular cell tumor; traumatic neuroma; oral traumatic neuroma; palisaded encapsulated neuroma; oral palisaded encapsulated neuroma.

## Introduction

Oral neural tumors (ONTs) may be either neoplastic or reactive in origin (1) and they arise from distinguishable compartments of peripheral nerves, which are the axons, endoneurium, perineurium and epineurium (2,3). The endoneurium comprises of fibroblasts, capillaries, macrophages and mast cells and surrounds axons which are formed by Schwann cells (2,3). The perineurium consists of perineurial cells arranged in one or more concentric layers with collagen fibers (4) while the epineurium is mainly comprised of fibro adipose tissue (2). ONTs are rare lesions (5-10). Jones and Franklin (6) reported 387 (0.88%) cases of benign and no case of malignant ONTs in patients over 16 years old, amongst 44,007 oral and maxillofacial pathology specimens submitted for histopathologic examination during a 30-year period. In patients younger than 16 years old, benign peripheral nerve sheath tumors comprised 0.7% and malignant neural tumors 0.02% of 4406 specimens of oral and maxillofacial pathology specimens submitted for histopathologic examination during a 30-year period (7). In a recent study (10), Alotaibi *et al.*, reported 340 (0.2%) cases of benign and no case of malignant ONTs amongst 164,578 specimens during a 22-year period.

The most common ONTs are neurofibroma (NF), traumatic neuroma (TN), schwannoma (SCH) or neurilemmoma and palisaded encapsulated neuroma (PEN) (5-13). However, in rare occasions other peripheral nerve sheath tumors may appear in the oral cavity. They may be benign, such as nerve sheath myxoma (NSM) (14), neurothekeoma (15) perineurioma (4), or malignant (16). The diagnosis of ONTs may be challenging due to overlap of histologic features and use of immunohistochemical markers may be essentials to set a definite diagnosis (11).

In the latest WHO classification of Head and Neck Tumors (17), oral granular cell tumor (GCT) is defined as an “uncommon benign tumor of Schwann- cell differentiation characterized by poorly demarcated accumulation of plump granular cells”. The schwannian differentiation can be justified by the intense reactivity for the immunohistochemical marker S-100 (17). However, Vered et al. (18), showed that oral GCT express a variety of immunohistochemical markers besides S-100 suggesting that they do not derive from a particular cell type. Additionally, few cases of oral GCT are S-100 negative (primitive polypoid granular cell tumors) (19,20). Nevertheless, following the latest WHO classification (17) and the opinion of other authors (5-11), oral GCT, will be considered an ONT in the present study.

The purpose of the present study is to describe the clinicopathological features of 157 ONTs and review the pertinent literature.

## Material and Methods

All cases of ONTs diagnosed from 1974 to 2017 were retrospectively retrieved from the files of the Department of Oral Medicine and Pathology. The diagnosis in each case was reconfirmed by studying representative 5μm - hematoxylin and eosin stained tissue sections. From the biopsy submission forms the following data were collected: age and gender of patients, site, clinical description, size, color, symptoms and duration of each lesion. All data were aggregated and tabulated using Microsoft Excel® 2016. The study was approved by the Research Ethics Committee of the Department of Dentistry, School of Health Sciences, National and Kapodistrian University of Athens, Greece (code number 371/11.07.2018).

## Results

Out of 35,590 biopsies accessioned during the study period, 157 cases of ONTs in equivalent number of patients were detected representing approximately 0.4% of the whole sample. The most common ONT was GCT (38.9%) followed by NF (19.7%), SCH (15.9%), TN (15.9%), PEN (8.3%) and NSM (1.3%). No case of malignant ONT was retrieved.

**Figure 1:**
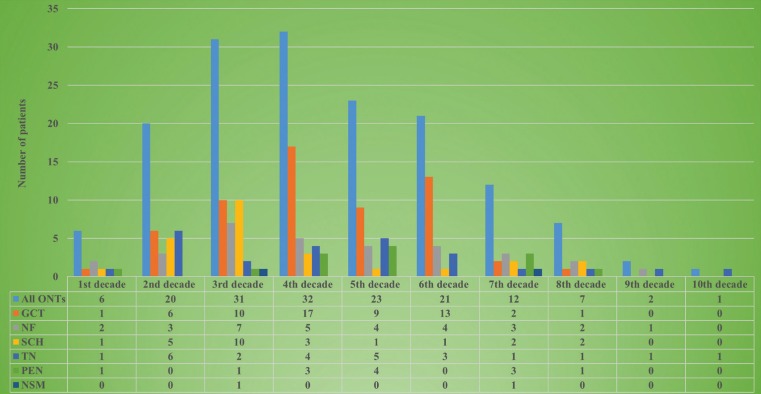
Number of patients in each decade of life for all ONTs.

Gender was recorded in 157 cases whereas age in 155. All demographic characteristics are tabulated in  Table 1 whereas the number of patients in each decade of life is illustrated in Figure 1. As a whole, ONTs had a slight female predominance with a male to female ratio of 0.8:1. The majority of patients were in the 3rd and 4th decade of life with a mean age of 38.4±18.8 years (median age: 36 years). Amongst ONTs, GCT, SCH and TN had the lowest male to female ratio whereas NF the highest. In addition, with the exceptions of NF, which occurred in younger female compared to male patients, all other neural tumors occurred in approximately the same aged patients, irrespectively of gender.

**Table 1:**
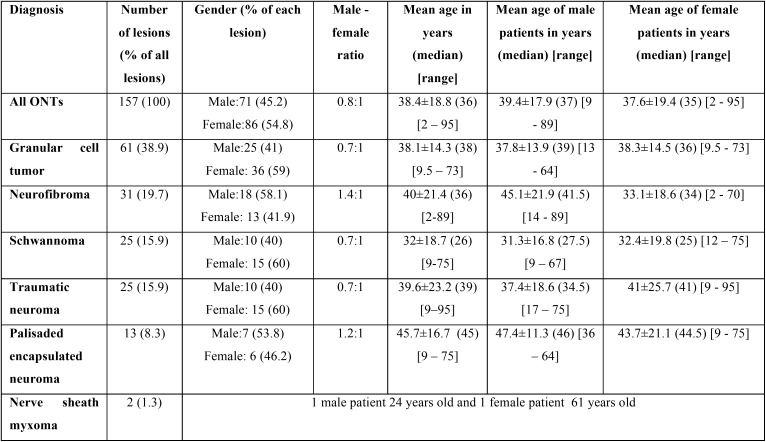
Demographic characteristics of 157 ONTs.

The exact location of lesions was recorded in 154 cases and has been tabulated in  Table 2. As a whole, ONTs more commonly occurred in the tongue (52.5%), followed in descending order by lip mucosa (12.3%) and hard palate (11.7%). GCT, SCH and TNM also more commonly occurred on the tongue. NF manifested with the same frequency in the tongue (23.3%) and hard palate (23.3%) whereas PEN in the hard palate (38.5%) and lip mucosa (30.8%).

**Table 2:**
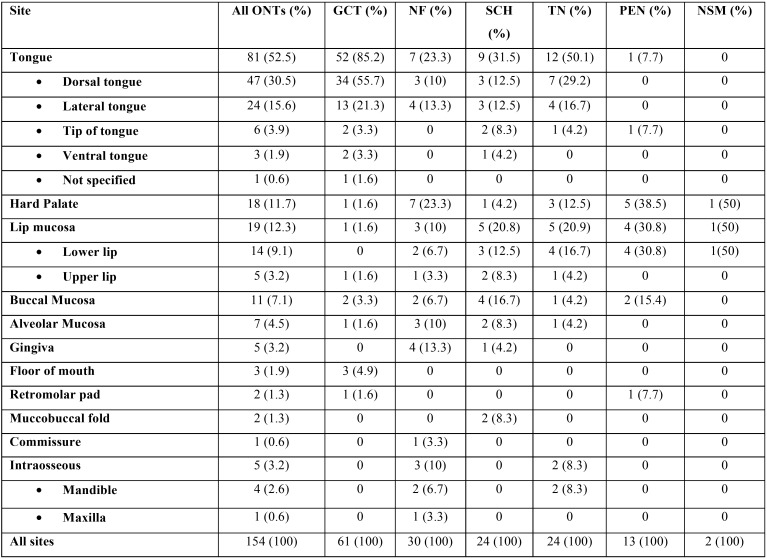
Location of 154 ONTs.

The clinical characteristics of all ONTs are tabulated in  Table 3. As a whole, ONTs were clinically described as nodules in 94.2% of cases. The majority of ONTs were of normal (58.6%) or white (29.3%) color, measuring 1±0.7 cm in their maximum diameter. The majority of lesions (85.6%) were asymptomatic thus justifying the long duration of 25±35.5 months. SCH had the greater size of all ONTs; NF was the ONT presenting more commonly with symptoms; GCT had the shortest and PEN the highest duration among ONTs of the sample. The clinical characteristics of ONTs are non-specific and mimic those of other oral lesions that is why the most common provisional diagnosis recorded was irritation fibroma (30.1%). The most common provisional diagnosis for each ONT was: GCT for GCT; irritation fibroma for NF, TN and PEN; lipoma and benign salivary gland tumor for SCH;.

**Table 3:**
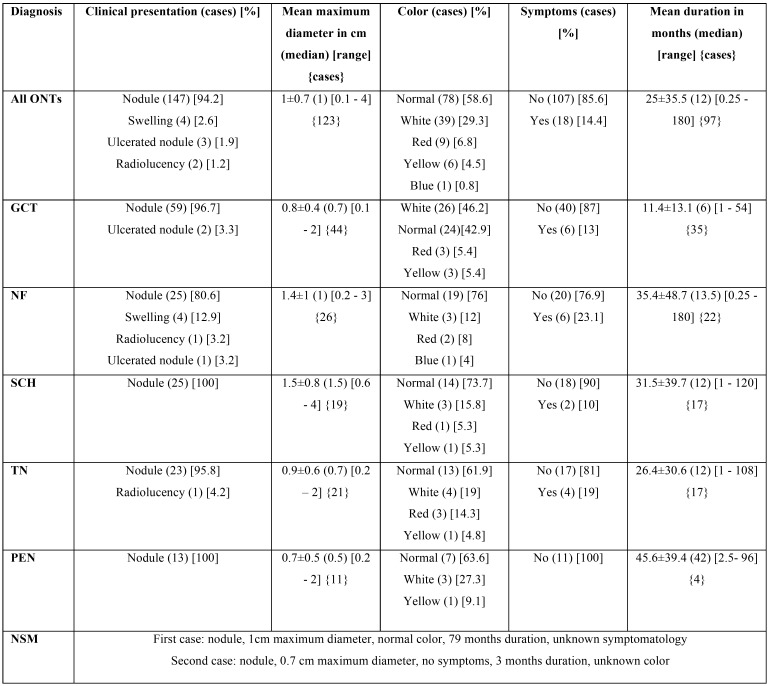
Clinical characteristics of 157 ONTs.

Concerning histopathological characteristics, pseudoepitheliomatous hyperplasia was noticed in 30 (49.2%) cases of GCT; 25 (80.6%) NFs were diagnosed as conventional, whereas 6 (19.4%) as plexiform (one plexiform NF arose on the upper lip mucosa of a 7.5-year-old female patient and was a manifestation of the already diagnosed neurofibromatosis type 1 (NF1) syndrome. We do not know if any of the other patients with plexiform NF at the time of diagnosis fulfilled the diagnostic criteria of NF1 since we acquired the data from the biopsy submission forms; 21 (84%) cases of SCH were classified as conventional, 2 (8%) cases as plexiform (We do not know if any of the patients with plexiform SCH at the time of diagnosis fulfilled the diagnostic criteria of neurofibromatosis type II (NF2) or schwannomatosis, since we acquired the data from the biopsy submission forms) and 2 (8%) as ancient; finally, 12 (92.3%) cases of PEN were classified as classic or lobular and 1 (7.7%) as plexiform.

## Discussion

In this retrospective study the demographic, clinical and histopathologic characteristics of 157 ONTs were described. In  Table 4, the number of cases of each ONT in our study as well as in similar ones of the literature (5-10), are tabulated. Upon combing the data of the studies of Table 4 that included GCT, NF, SCH, TN and PEN on their sample (current study, 6-9), ONTs represent 0.7% of all specimens from the oral and maxillofacial area submitted for histopathologic examination and the most common ONT is NF (38.2%) followed by TN (30%), GCT (15.4%), SCH (12.2%), PEN (2.8%), other benign ONT (0.9%) and finally malignant peripheral nerve sheath tumors (0.4%). In our study, GCT was the most common ONT (38.9%) followed by NF (19.7%). As can be easily noticed, there are many minor differences concerning the frequency of each ONT amongst the different studies included in Table 4 that can be probably explained by the differences in the number of patients included and the country of origin of each study (10).

**Table 4:**
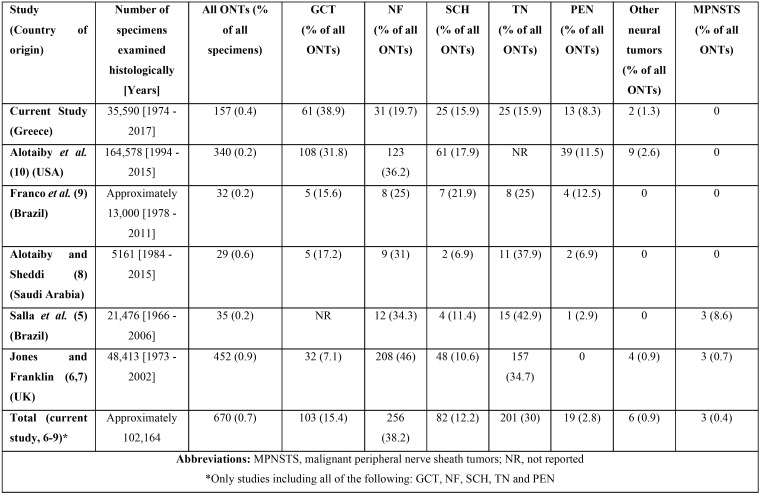
Studies of the literature concerning ONTs.

In the following paragraphs, a review of the literature concerning ONTs found in our department will be displayed.

-Neurofibroma

NF is less prevalent than schwannoma in the head and neck area, whereas the opposite applies when they occur intraorally (21). It is the first (6,7,9,10) or second (5,8, current study) most common ONT. In the majority of cases, it arises in soft tissues, while it is very rare to appear in the jaws (22). It usually appear in the palate, gingiva and tongue (5,11) and clinically presents as solitary and less frequently as multiple asymptomatic small sessile nodules covered by normal appearing mucosa (21).

5% (10) – 25% (21) of oral NF arise in the setting of NF1. A suspicion of NF1 may be raised when multiple NFs are noticed in the oral cavity or when NF is of the plexiform variant (21). However multiple or plexiform NFs may arise in patients without NF1 (23,24) NF1 (OMIM #162200) is an autosomal dominant multisystem disorder caused by heterozygous mutations in the neurofibromin gene on chromosome 17q11.2 (25). In order to set a diagnosis of NF1, any two out of the following criteria must be met (26):

1. Six or more café-au-lait macules (with a greatest diameter of >5 mm in pre-pubertal subjects, or <15 mm in post-pubertal subjects)

2. Two or more NFs of any type or one plexiform neurofibroma

3. Freckling in the axillary or inguinal region

4. Optic glioma

5. Two or more Lisch nodules (iris hamartomas)

6. A distinctive osseous lesion such as sphenoid dysplasia or thinning of long bone cortex with or without pseudoarthrosis

7. A first-degree relative with NF1

Patients with NF1 may have one or more of the following osseous, oral and dental features apart from NF: short maxilla or mandible or cranial base, macrocephaly, low face height, enlargement of the mandibular canal, more frequent Class III dental malocclusions, alterations in the temporomandibular joint, gingival enlargement, gingiva pigmentation, macroglossia, enlargement of fungiform papillae, impacted, supernumerary, missing, or displaced teeth and periapical cementum dysplasia (25,27). While NFs in patients with NF1 have a greater chance of malignant transformation, this has rarely been reported for oral or perioral lesions (27).

Histopathologically, conventional NF is circumscribed but non-encapsulated and composed of randomly and uniform distributed spindle cells with wavy or serpiginous and hyperchromatic nuclei, in a loose collagenous stroma, which is often myxoid (1,11). Another characteristic feature is the presence of significant amount of mast cells (12). Intraneural NF, has the same microscopic features as conventional neurofibroma, while its stroma is more collagenous and resembles “shredded carrots” (12). Plexiform NF consists of expanded tortuous nerve branches and bundles of wavy, spindle cells (24). In the study of Alotaibi *et al.* (10), 5% of NFs were of the plexiform variant, whereas in the present study they represented 19.4% of all neurofibromas.

 Rare histologic variants of NF described in the oral cavity are NF with prominent differentiation of Meissner bodies (28) and the very rare dendritic cell NF with pseudorosettes (29).

With the use of silver stains, nerve axons can be visualized inside the tumor’s stroma (11). Cells in NF are S-100 (Schwann cells) positive but not in the same extent as SCH (11). Some of them are also positive for EMA (perineurial cells), CD34 (endoneurial fibroblasts), type IV collagen (expressed by basement membranes of nerve sheath cells) and CD68 (putative resident macrophages) (11). Salla *et al.* (1) have also shown that in approximately 80% of cases, cells of NF are GLUT-1 (perineurial cells) positive, while Koutlas and Scheithauer (12) mention that cells stain positively but in varying degree for GFAP. From the above it is concluded that cells in NF are a mixture of Schwann cells, perineurial cells and endoneurial fibroblasts, and the proportion of each type of cell, varies amongst different tumors as well as in different areas of the same tumor (11).

-Schwannoma

Head and neck is the most common site where SCH arise (30,31). In the oral cavity, however they are not as common as they represent 9.3% (30) – 13.5% (32) of all head and neck SCHs. SCH is the third (5-7,9,10,current study) or fourth (8) most common ONT. Most commonly, it presents as a peripheral lesion, but rarely it may arise inside the jawbones (10,33). Its most common sites of manifestation are the lips and specifically the lower lip (10,11,31) or the buccal mucosa (5,34). Clinically, oral SCHs manifest as slow-growing solitary masses covered by normal appearing mucosa that measure a few millimeters to many centimeters in diameter (31). Most cases are asymptomatic, but pain has been reported in up to 1/3 of the cases (30). In rare cases, difficulty in swallowing, speaking or breathing, bleeding, macroglossia, or loss of sensation and taste may be mentioned by patients (31,35,36).

Microscopically, conventional SCHs are usually encapsulated tumors (11,30) demonstrating a proliferation of cells that are organized in two different architectural tissue patterns (11,13,33,34): the most common Antoni A, characterized by fascicles of spindle-shaped cells streaming around acellular eosinophilic areas known as Verocay bodies, and the less cellular and less organized Antoni B, where spindle cells are randomly arranged in a loose myxomatous stroma. In Antoni B areas mast cells may be found (12). In the majority of cases, Antoni A pattern prevails (11,30) and the term cellular SCH has been used by some authors when the tumors is composed predominantly or exclusively by Antoni A areas without Verocay bodies formation (33). Other possible microscopic finding are hyalinized and/or ectatic vessels that may be accompanied by hemosiderin deposits and degenerative changes most commonly in lost-standing lesions such as hemorrhage, infraction, dystrophic calcifications, cyst formation, nuclear atypia and hyperchroamasia (13,34,37). When these degenerative changes are noticed, the SCH is termed ancient (37). However, these changes are less frequently noticed in oral SCHs compared to tumors in other parts of the head and neck area (30). Another histologic variant is plexiform SCH (13), which is characterized grossly and microscopically by a multinodular, plexiform pattern, with all the essential features of conventional SCH. Alotaibi *et al.* (10) reported that 5% of the SCHs of their study were of the ancient and 1.6% of the plexiform variant whereas in the present study each of those two histologic variants accounted for 8% of all SCHs. Other rare histologic variants of intraoral SCH include SCH with pseudoglandular elements (38), melanotic SCH (39), SCH with epithelial induction (40) and neuroblastoma – like SCH (41).

Neural axons may be noticed in the surrounding connective tissue and not inside the lesion (11). The cells in SCH are S-100 and collagen IV positive and the staining is greater in Antoni A compared to Antoni B areas (11). The cells are also variably positive for CD68; EMA stains only the capsular tissues and not the lesion’s cells; CD34 stains Antoni B areas as well as capsular tissues whereas Antoni A areas are CD34 negative (11). The capsule also stains positive for GLUT-1 (1) and lesional cells in approximately 50% of cases are GFAP positive (12). The EMA/GLUT-1 positivity of the capsule implies that it originates from the perineurium (1), whereas the diffuse stains of lesional cells for S-100 suggest they are Schwann cells (11).

Two hereditary syndromes that are related to SCH are NF2 and schwannomatosis (13). Oral SCHs are rarely associated with these syndromes (42), and the likelihood of having either one is greater when the plexiform variant is diagnosed (13). NF2 (OMIM #101000) is an autosomal dominant disorder caused by mutation of the NF2 gene on chromosome 22q12.2 (43). Clinically, it is characterized by the development of vestibular schwannomas; other cranial, spinal or cutaneous nerve schwannomas; cranial and spinal meningiomas or other central nervous system tumors; ocular abnormalities (early onset cataracts, optic nerve sheath meningiomas, retinal or pigment epithelial hamartomas or both and epithelial retinal membranes); skin abnormalities (subcutaneous schwannomas, and café au lait pigmentation) (43). A patient must fulfill at least one of the following five criteria, in order for NF2 to be diagnosed (44):

1. Bilateral vestibular SCHs in patients <70 years

2. First degree relative family history of NF2 and unilateral vestibular SCH in patients <70years

3. First degree relative family history of NF2 or unilateral vestibular SCH and 2 of meningioma, cataract, glioma, neurofibroma, SCH, cerebral calcification (if unilateral vestibular SCH + ≥ 2nonintradermal SCHs, need negative LZTR1 gene test)

4. Multiple meningiomas (2 or more) and 2 of unilateral vestibular SCH, cataract, glioma, neurofibroma, SCH, cerebral calcification 

5. Constitutional or mosaic pathogenic NF2 gene mutation in blood or identical mutations in 2 distinct tumors

Schwannomatosis (OMIM #162091) is a rare disorder characterized by predisposition to develop multiple SCHs (that commonly affect peripheral nerves and the spine) and less commonly meningiomas, while the most common symptom is chronic pain, either diffuse or local (45). The majority of schwannomatosis’ cases are sporadic with only 13%-25% being familial (45). Until now two predisposition genes for schwannomatosis have been identified, SMARCB1 on chromosome 22q11.23 and LZTR1 on chromosome 22q11.21 (45). Diagnosis can be set with the combination of both molecular and clinical testing or be only clinical (45):

1. Combined molecular and clinical diagnosis: ≥2 tumors with loss of heterozygosity on the long arm of chromosome 22 and 2 different somatic NF2 mutations and ≥2pathologically confirmed SCHs or meningiomas or germline SMARCB1 or LZTR1 pathogenic mutation and one pathologically confirmed SCH or meningioma

2. Clinical diagnosis: ≥2 non-intradermal SCHs, one pathologically confirmed and no bilateral vestibular SCHs by high quality MRI (some mosaic NF2 patients will be included in the diagnosis at a young age and some schwannomatosis patients may have unilateral vestibular SCHs or multiple meningiomas) or one pathologically confirmed SCH or intracranial meningioma and an affected first degree relative

3. Exclusion criteria for schwannomatosis include at least one of the following: germline pathologic NF2 mutation; diagnostic criteria for NF2 fulfilled; first degree relative with NF2; schwannomas occurring in a region of previous radiation therapy.

If upon microscopic examination a hybrid tumor (having characteristics of both NF and SCH) or an abundant myxoid stroma are noticed, NF2 or schwannomatosis should be suspected (46). However, in 22.2% of oral SCHs not associated with any syndrome, areas resembling NF may also be noticed (30).

-Traumatic Neuroma

TN is the first (5,8,9), second (6,7) or third (current study) most frequent ONT. It is a reactive lesion (1) and represents a copious growth response to nerve injury (47). The majority of TNs are peripheral and only a few cases of intraosseous TNs have been reported in the English literature (48). Chrysomali et al. (11) reported that 9.5% of TNs were intraosseous, whereas in the study of Peszkowski and Larsson (49), 24.4% of oral TNs were located inside the jaw bones. In our Department, only 8.3% of TNs were intraosseous. Clinically, TN most commonly manifests as non-ulcerated nodule with smooth surface (48) on the tongue, mental foramen and lip mucosa (5,11). In 25% - 30% of cases, oral TNs are painful (48). In our study, TNs, 19% of TNs were painful while the ONT presenting more often with symptoms was NF.

Histopathologically, TN is non encapsulated (12) and characterized by numerous bundles of axons, nerve fibers and Schwann cells in a dense fibrous connective tissue stroma (1,11). The connective tissue may be mucoid and its nature depends on the age of the lesion (12). Sometimes ganglion cells may also be noticed (47). With the use of silver stain, many axons having parallel distribution can be identified inside nerve fascicles (11). Immunohistochemically, cells inside nerve fascicles are S-100 positive and more than 50% of them reacts positively for CD57 (myelin-associated glycoprotein) (11). The only other ONT that reacts positively for CD57 is dendritic cell NF with pseudorosettes (29). EMA stains more than 50% of cells in the perineurium, CD34 stains cells of the endoneurium (11) and GLUT-1 is always positive on perineurial cells (1).

-Granular cell tumor

The majority of GCTs manifest in the head and neck area with the oral cavity and specifically the tongue being the most prevalent site (10,18,50,51). Oral GCT is the first (current study), second (10), third (8) or fourth (6,7,9) most common ONT.

Microscopically GCT in the majority of cases (57%) is poorly circumscribed and constitutes of round, oval, polygonal or spindle shaped granular cells with dark or vesicular nuclei arranged in sheets, ribbons or islands separated by septa of fibrous tissue (18). The granular cells may involve or replace the muscle fibers whereas it is not common to envelope nerve bundles (18). A common finding in GCT is pseudoepitheliomatous hyperplasia (PEH). Alotaibi *et al.* (10) found PEH in 6.5% of GCTs, Vered et al. (18) in 25% and in our study PEH was recorded in 49.2% of GCT cases. According to Vered *et al.* (18), the difference in the reported rates of PEH can probably be attributed to the definition of PEH adopted from each author. Immunohistochemistry is not necessary for the diagnosis of GCT, as the histopathologic findings are pathognomonic. Granular cells are positive for S-100 in the majority of cases (18,19). However when S-100 immunostaining is negative, then the lesion, as mentioned in the introduction, is termed “primitive polypoidGCT” and is characterized by greater cytologic atypia and higher mitotic index compared to granular cell tumor (20).

In 1998, Fanburg-Smith *et al.* (52), proposed that GCTs fulfilling at least three of the following histopathologic criteria should be classified as malignant: nuclear pleomorphism; tumor cell spindling; vesicular nuclei with large nucleoli; increased nuclear:cytoplasm ratio; necrosis; increased mitotic rate (>2 mitoses/10HPF). If one or two of the above criteria were fulfilled, GCTs should be classified as atypical and benign when none of the above criteria presented during microscopic examination (52). In 2011, Nasser *et al.* (53), in an attempt to simplify the diagnostic criteria of malignant GCT, proposed that when necrosis and/or increased mitotic rate (>2 mitoses/10HPF) were present, GCTs had an uncertain malignant potential, whereas when none of the two existed microscopically, GCTs were considered benign. Nevertheless, because GCTs without atypical histological features have showed malignant behavior, Machado *et al.* (54) proposed that metastasis is the only definite criterion of malignancy in GCT and that lesions with atypical histological features should be termed “with increased risk of metastasis” and not “malignant”. In cases of multiple GCTs, benign histology favors the possibility of multiple rather than malignant lesions with the exception of occurrence in the lymph nodes, bones, lungs or liver where malignant GCT should be suspected despite the lack of atypical histologic features (54). The same applies for metachronous lesions (50). To the best of our knowledge, no case of malignant GCT has been reported in the oral cavity.

-Palisaded encapsulated neuroma

PEN (or solitary circumscribed neuroma), is the least frequent ONT (5,8-10, current study). Clinically it present as small painless nodule more commonly on the palate, gingiva and lip mucosa (12). Microscopically, it is often mistaken with SCH and less frequently with NF as reported by Jordan *et al.* who after reviewing slides of older cases, reclassified 12 SCHs and 4 NFs as PENs (55). It can be classified as classic or lobular, which is the more frequent histologic variant, or plexiform, fungating and multilobular (12). These histologic variations are of neither clinically or biologically significance (12). In the majority of cases, palisaded encapsulated neuroma is well circumscribed but non-encapsulated (12). Cells are spindle and arranged in fascicles that show tendency for palisading (11). These fascicles may blend with the connective tissue immediately subjacent to the epithelium (12). A characteristic finding is the frequent artificial clefting due to tissue shrinkage (12). The stroma is collagenized and rarely mucoid and mast cells may be evident in some lesions (12). Axons are present in variable amounts and patterns and are better visualized with special stains (11,12)

The cells react positively with S-100 and always negative for GFAP (12). EMA in peritumoral tissue in inconsistently expressed (11,12) and stains no more than one layer of cells (11). Some tumoral cells also stain positive for CD34 (11).

-Nerve sheath myxoma and neurothekeoma

Oral NSM is a rare ONT. In our Department we found only two cases of NSM, one of which has been already published (56). In a systematic review by Rozza-de-Menezes et al. (14), only 25 cases of NSM could be retrieved until 2012. It has a slight female predilection and the mean age of patients is 35.9 years, with the majority of cases diagnosed in the 4th and 5th decade of life (14). Clinically, in the majority of cases, it presents as an asymptomatic firm nodule of normal color on the gingiva, buccal mucosa or tongue (14). Histologically, NSM is well circumscribed but non-encapsulated, and characterized by myxoid nodules of varying shape and size, separated by dense collagenous septa (14,57). The myxoid matrix is composed of hyaluronic acid as it reacts positive in Alcian Blue staining at pH 2.5 (14). The cells have a small, ovoid and bland nuclei and are arranged in such pattern that give nodules a lamellated or whorled appearance (57). In rare instances, cells may have their nuclei located in the periphery of the cytoplasmic membrane or exhibit multinucleation (57). Also, rare histological features are epithlelioid morphology of cells, nuclear palisading, Verocay – like formations and cystic degeneration (58). Mast cells are frequently seen (14). Immunohistochemically, cells react positively for S-100, S-100A6 and NSE (non – specific enolase) while variably for GFAP, CD57 and NGFR (nerve growth factor receptor) (14,57). Cells do not react for EMA (14).

Neurothekeoma is a rare lesion that histologically mimicks oral NSM (14,57,58). Some authors use the term “classic neurothekeoma” for NSM and “cellular neurothekeoma” for neurothekeoma (58). However, by using microarray analysis, Sheth *et al.* (59), showed that NSM is genetically closer to SCH while neurothekeoma to fibrous histiocytoma. Oral neurothekeoma is a very rare lesion with less than ten cases reported (58,60). Clinically, in the majority of cases, oral neurothekeoma has a female predominance, patients are in the 2nd or 3rd decade of life and clinically presents as an asymptomatic or mildly painful firm nodule of normal color in the tongue or gingiva (58,60). Histologically, neurothekeoma is non-encapsulated and poorly circumscribed (57) and consists of epithelioid to spindle cells organized in nests or bundles separated by dense collagen (58). These nests are smaller and more uniformly sized compared to the one of nerve sheath myxoma (57). In some cases cells may exhibit atypia and/or mitotic activity and the nests or stoma may be myxoid (57). There is also a single report of desmoplastic variant of neurothekeoma is the oral cavity which is characterized by marked stroma hyalinization (60). Immunohistochemically, cells react positive for S100A6 and NSE and negative for S-100 (57,58). Vered *et al.* (58), showed that cells of neurothekeoma react positively for NKI/C3 while the opposite stands for nerve sheath myxoma. Plexiform NF may also stain positive for NKI/C3 but less intensely than neurothekeoma (58).

## Conclusions

Tumors of neural origin are rare in the oral cavity as confirmed in this study. They have a female predominance, they most commonly occur in the 3rd and 4th decade of life and the most common site of occurrence is the tongue. Their clinical presentation mimic other common oral lesions and thus microscopic examination is the only mean to arrive at a definitive diagnosis.
